# High-Performance Carbon Fiber Paper Enabled by Amino Resin-Derived Low-Temperature Carbonization

**DOI:** 10.3390/ma19061230

**Published:** 2026-03-20

**Authors:** Tao Qin, Xiaosong Pu, Shouqing Liu, Taohong Li, Shuyang Jiang, Xuemei Li

**Affiliations:** 1 College of Materials and Chemical Engineering, Southwest Forestry University, Kunming 650224, China; qintao@swfu.edu.cn (T.Q.); pxsxnlydx@163.com (X.P.); sqliu@swfu.edu.cn (S.L.); syjiang@swfu.edu.cn (S.J.); 2 International Joint Research Center for Biomass Materials, Southwest Forestry University, Kunming 650224, China

**Keywords:** amino resin, carbon fiber paper, low-temperature carbonization, nitrogen doping

## Abstract

Conventional phenolic-resin-based carbon fiber paper (CFP) typically suffers from low mechanical strength, poor toughness, insufficient pore interconnectivity, and a reliance on extreme high-temperature graphitization to attain high conductivity. This study employs a novel melamine-hexamethylenediamine (MH) thermosetting resin as the binder to fabricate MH resin-based CFP (MHCFP). Through the synergistic effects of robust interfacial bonding, triazine-ring-induced low-temperature formation of sp^2^ carbon clusters, and nitrogen doping, the MHCFP achieves comprehensive performance superiority over the phenol-formaldehyde (PF)-based CFP (PFCFP) at moderate carbonization temperatures (500–700 °C): MHCFP exhibits superior toughness, tensile strengths of 23–45 MPa (vs. PFCFP’s 8–18 MPa), and in-plane resistivity of 24–39 mΩ·cm (vs. PFCFP’s 54–83 mΩ·cm). Furthermore, MHCFP possesses a highly open macroporous structure (porosity > 78%), ensuring excellent gas permeability and water management capability. This work presents a promising low-temperature strategy for developing high-performance CFP, showing great potential for next-generation proton exchange membrane fuel cell gas diffusion layers.

## 1. Introduction

Carbon fiber paper (CFP) is widely used in electrochemical energy devices (e.g., gas diffusion layers for proton exchange membrane fuel cells) due to its porous structure, excellent electrical and thermal conductivity, as well as good processability, thermal stability, and chemical resistance [1,2,3,4,5]. Currently, industrial production commonly adopts a fabrication process involving impregnation, hot-pressing, and carbonization. These processes facilitate the formation of a carbonaceous bonding phase through the pyrolysis of organic adhesives, thereby securely integrating carbon fibers into a stable three-dimensional network [6,7,8,9]. In addition to this mainstream route, researchers have also investigated an alternative route involving the incorporation of polyphenylene sulfide fibers during the preforming stage for binder-free consolidation via thermal welding [10]. However, this approach faces limitations, including high cost [11], and has not yet replaced the established resin pyrolysis-based carbon bonding process in scaled-up production. Given these limitations, the preparation route based on organic adhesive pyrolysis carbon bonding offers advantages, including relative process simplicity, controllable cost, and high reliability, and therefore remains the predominant technology in the current industrial production of the CFP [12,13,14,15].

Currently, alcohol-soluble thermosetting phenolic resin has become the most widely adopted binder in CFP fabrication, owing to its high char yield, low cost, and industrial maturity [16,17,18,19]. However, studies indicate that such phenolic resins exhibit weak interfacial adhesion with carbon fibers and tend to generate numerous microcracks within the CFP, resulting in stress concentration. This leads to pronounced brittleness and inferior mechanical properties in the fabricated CFP [20,21,22,23], significantly limiting its practical applicability. To address these issues, researchers have attempted to improve carbon paper performance by modifying phenolic resin or incorporating functional components such as graphite [24,25,26,27,28,29]. Although these modifications have improved certain properties to some extent, achieving satisfactory electrical performance still necessitates graphitization treatment at temperatures of 2000 °C or above [30,31,32]. Consequently, it remains a considerable challenge to simultaneously attain high mechanical strength and excellent electrical conductivity in the CFP at relatively low carbonization temperatures.

In this study, we adopted a novel amino resin (MH) independently developed by our team as the binder for carbon fiber paper fabrication. This resin features abundant triazine ring structures and nitrogen atoms, leading us to hypothesize that: (1) the triazine-ring planar structure would direct the low-temperature formation of sp^2^ carbon clusters, while amine/amide nitrogen dopants would tune the electronic structure of the carbon network and introduce additional charge carriers, collectively establishing an efficient charge-transport pathway; and (2) the MH resin would form strong interfacial bonding with the carbon fiber surface, effectively suppressing interfacial debonding during carbonization. These hypotheses were subsequently validated through systematic characterization and performance testing, demonstrating that the MH-resin-based CFP (MHCFP) demonstrates outstanding comprehensive properties even at low carbonization temperatures (500–700 °C): (1) excellent mechanical performance—tensile strength reaches 23–45 MPa, far exceeding that of PFCFP (8–18 MPa) treated under identical conditions, accompanied by remarkable toughness; (2) outstanding electrical conductivity—the in-plane resistivity of MHCFP (24–39 mΩ·cm) is significantly lower than that of PFCFP (54–83 mΩ·cm) processed under the same regime; (3) high hydrophobicity—the water contact angle of MHCFP (125–127°) remains consistently higher than that of PFCFP (123–125°); (4) an ideal transport structure—porosity up to 78.8% with pore sizes concentrated in the macroporous range of 10–100 μm, forming a highly interconnected three-dimensional network that structurally ensures excellent gas permeability and water management. Briefly, this study highlights the significant advantages of MH resin as a CFP binder for fabricating CFP at low temperatures that integrates high strength, high conductivity, and high permeability.

## 2. Materials and Methods

### 2.1. Materials

Melamine (M, AR) and hexamethylenediamine (H, AR) were purchased from Chengdu Chron Chemicals Co., Ltd. (Chengdu, China). Ammonium chloride (NH_4_Cl, AR), sodium carboxymethyl cellulose (Na-CMC), and anhydrous ethanol (C_2_H_5_OH, AR) were obtained from Sinopharm Chemical Reagent Co., Ltd. (Shanghai, China). Phenolic resin, as the comparative binder, was sourced from online suppliers. All chemicals were used as received without further purification.

### 2.2. Synthesis of MH Resin

The MH resin was synthesized via a one-pot method according to our previous study [33]. Briefly, melamine (M) and hexamethylenediamine (H) at a molar ratio of 1:2 were added into a three-neck flask, followed by the addition of NH_4_Cl as a catalyst (6 wt% relative to melamine). The reaction mixture was heated in an oil bath at 200 °C under continuous stirring. During the reaction, the released ammonia (NH_3_) was absorbed by a tail-gas absorption setup. The heating was stopped when the mixture turned colorless and transparent. The product was then cooled to room temperature to obtain the MH resin.

### 2.3. Preparation of the Carbon-Fiber Preform Paper

**Figure 1:**
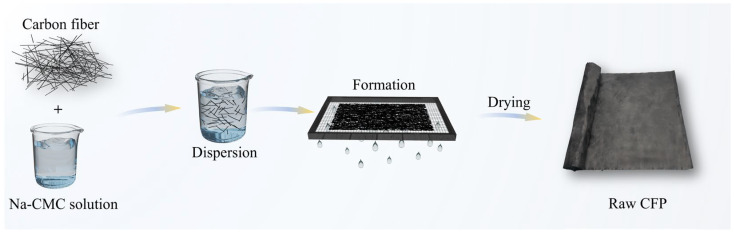
Schematic illustration of the preparation process for the carbon-fiber preform paper. Abbreviations: Na-CMC, sodium carboxymethyl cellulose.

The carbon-fiber preform paper was fabricated via a conventional wet-lay process (Figure 1) [34]. Briefly, chopped carbon fibers were uniformly dispersed in an aqueous solution containing a dispersant (1‰ in mass fraction). The resulting suspension was then transferred to a wet-lay forming unit, where the fibers were collected and consolidated by rapid filtration. After drying, the obtained sheet was denoted as Raw CFP.

### 2.4. Fabrication of CFP

The MH solutions with different concentrations were prepared using a 70% ethanol aqueous solution. A carbon fiber preform sheet (12 cm × 16 cm) was impregnated in the MH solution for 1 min and then dried in an oven at 120 °C for 15 min to evaporate the solvent. Subsequently, the dried preform was hot-pressed in a platen vulcanizer. The hot-pressing protocol was as follows: pre-heating at 240 °C for 3 min, then pressurizing to 0.2 MPa and holding for 5 min, followed by ramping the temperature to 270 °C at a rate of 2 °C/min while gradually increasing the pressure to 4 MPa and maintaining these conditions for 40 min; finally, the system was cooled naturally to room temperature under pressure. The resulting material is denoted as HP-MHCFP. For systematic comparison, a control sample was prepared using the same impregnation process but with a thermosetting phenolic resin as the binder, denoted as HP-PFCFP.

The as-prepared HP-CFP samples were placed in a tube furnace and carbonized under a nitrogen atmosphere. The carbonization protocol was as follows: ramping to 400 °C at 10 °C/min, holding for 120 min, then ramping the temperature to the target final temperature at 2 °C/min and maintaining for 60 or 120 min. The resulting samples were denoted as CFP. Based on the maximum decomposition temperature of MH resin being approximately 500 °C [33], four carbonization temperatures—500 °C, 600 °C, 700 °C, and 900 °C—were investigated in this study. The corresponding samples were designated as CFP-500, CFP-600, CFP-700, and CFP-900.

### 2.5. Thermogravimetric Analysis

Thermogravimetric analysis was performed using an STA 2500 simultaneous thermal analyzer (NETZSCH, Selb, Germany). The measurement was conducted under a nitrogen atmosphere with a heating rate of 10 °C/min from 30 °C to 910 °C.

### 2.6. Scanning Electron Microscopy (SEM) Characterizations

The microstructure and surface elemental composition of the samples were characterized using a MIRALMS scanning electron microscope (TESCAN, Brno, Czech Republic) equipped with an OXFORD X-ray energy dispersive spectrometer (Oxford Instruments, Abingdon, UK). Prior to imaging, the samples were sputter-coated with a thin layer of platinum to enhance conductivity. Secondary electron images were acquired at an accelerating voltage of 10 kV. Elemental composition analysis was performed via energy-dispersive spectrometer scanning over selected areas.

### 2.7. X-Ray Diffraction (XRD) Characterizations

The crystal structure characteristics of the CFPs were determined using an Ultima IV X-ray diffractometer (Rigaku, Tokyo, Japan). The measurement conditions were as follows: Cu Kα radiation (λ = 1.5406 nm), diffraction angle (2θ) range of 10–80°, scanning speed of 5°/min, tube voltage of 40 kV, and tube current of 30 mA. Peak fitting was performed on the obtained XRD patterns to calculate the microcrystalline parameters of the CFP. The interlayer spacing d_002_ and the stacking thickness L_c_ of the graphite-like crystallites were calculated using the following Equations (1) and (2):

(1) $$d_{002}=\frac{\lambda }{2sin\theta }$$

(2) $$L_{c}=\frac{0.89\lambda }{\beta cos\theta }$$

$d_{002}$ is the interlayer spacing of graphite-like crystallites in the CFP (nm), λ is the wavelength of the incident X-rays (nm), θ is the Bragg angle (in radians), and $L_{c}$ is the stacking thickness of graphite-like crystallites (nm). β is the full width at half maximum (FWHM, in radians of theta) [35].

### 2.8. X-Ray Photoelectron Spectroscopy (XPS) Characterizations

The surface elemental composition and chemical states of the CFPs were analyzed using a K-Alpha X-ray photoelectron spectrometer (Thermo Scientific, Waltham, MA, USA). The measurement conditions were as follows: analysis chamber pressure of 2 × 10^−5^ Pa, monochromated Al Kα X-ray source (hv = 1486.6 eV), accelerating voltage of 12 kV, emission current of 6 mA, output power of 72 W, and a spot diameter of approximately 400 μm. Survey spectra were acquired in constant analyzer energy (CAE) mode with a pass energy of 100 eV, while high-resolution spectra were recorded with a pass energy of 30 eV. All spectra were calibrated using the C 1s peak at 284.8 eV as a reference.

### 2.9. Raman Characterization

The structural characteristics and degree of graphitization of the CFPs were analyzed using an Alpha300R high-resolution confocal Raman microscope (WITec, Ulm, Germany). The measurements were performed under the following conditions: laser wavelength 532 nm, laser power 8.0 mW, 300 g/mm grating, Olympus 20× objective (NA 0.25) (Olympus, Tokyo, Japan), integration time 40 s, and 6 accumulations. The Raman spectra were fitted by peak deconvolution to obtain the intensities of the G-band and D-band. The graphite molar fraction and crystallite size were then calculated using the following Formulas (3) and (4):

(3) $$X_{G}=\frac{I_{G}}{I_{G+I_{D}}}\times 100\%$$

(4) $$L_{a}=\frac{4.35}{I_{D}/I_{G}}$$

$X_{G}$ is the graphite molar fraction (%), $I_{G}$ and $I_{D}$ denote the intensities of the G-band and D-band, respectively (a.u.), $L_{a}$ is the in-plane crystallite size of the graphite crystallites in the CFP (nm) [36].

### 2.10. Thickness Measurement

The thickness of the CFPs was measured using a SAY-21 benchtop thickness gauge (Yueqing Huaxia Measurement Instrument Co., Ltd., Yueqing, China). Five measurement points were taken for each sample, and the recorded thickness values were averaged to obtain the final result.

### 2.11. Areal Density Measurement

The areal density was determined in accordance with GB/T 20042.7-2024 [37]. Five specimens with dimensions of 16 cm × 12 cm were cut from the prepared CFP. The mass of each specimen was weighed using a YH-M6002 analytical electronic balance (Yongkang Wu Xin Weighing Equipment Co., Ltd., Yongkang, China). The areal density was calculated using the following Formula (5):

(5) $$\rho_{A}=\frac{m}{A}$$

$\rho_{A}$ is the areal density (mg/cm^2^), m is the mass of the specimen (mg), and A is the area of the specimen (cm^2^).

### 2.12. Determination of Resin Char Yield

The char yield of the resin was measured using a TG 209 F1 thermogravimetric analyzer (NETZSCH, Selb, Germany). Under a nitrogen atmosphere, the temperature was first increased to 400 °C at a heating rate of 10 °C/min and held for 120 min. The initial mass of the resin ($m_{0}$) was recorded. Subsequently, the temperature was raised to the target temperature at a rate of 2 °C/min and maintained for 60 or 120 min. After natural cooling to room temperature, the final mass ($m_{f}$) was recorded. The char yield (***CY***) was calculated according to the following Formula (6):

(6) $$CY=\frac{m_{f}}{m_{0}}\times 100\%$$

### 2.13. Tests of Tensile Properties

Tensile properties tests were performed on a HK-318 universal servo mechanical testing machine (Dongguan Huakai Testing Equipment Technology Co., Ltd., Dongguan, China) in accordance with the GB/T 20042.7-2024 [37]. Specimens with dimensions of 10 mm × 80 mm were tested at a crosshead speed of 1 mm/min. Five parallel tests were conducted for each sample. Tensile strength (σ) and strain (ε) were calculated using the following Formulas (7) and (8):

(7) $$\sigma =\frac{P}{b\times h}$$

(8) $$\varepsilon =\frac{L-L_{0}}{L_{0}}\times 100\%$$

σ is the tensile strength (MPa), P is the maximum load (N), b is the width of the specimen (mm), h is the thickness of the specimen (mm), ε is the strain (%), $L_{0}$ is the initial gauge length (mm), and L is the gauge length at fracture (mm).

### 2.14. In-Plane Resistivity Tests

The in-plane resistivity of the CFPs was measured using an ST2258C multifunctional digital tester (Suzhou Jingge Electronics Co., Ltd., Suzhou, China) equipped with an ST2558B-F01 four-point probe. Measurements were performed at five distinct locations, including both peripheral and central regions of the sample, and the reported value represents the mean of these measurements.

### 2.15. Water Contact Angle Measurements

The water contact angle was determined using an SDC-100S optical contact angle goniometer (Dongguan Sindin Precision Instrument Co., Ltd., Dongguan, China). For each sample, five measurements were conducted at different surface positions, and the average value was calculated as the final result.

### 2.16. Mercury Intrusion Porosimetry

The pore structure of CFPs was quantitatively characterized using an AutoPore V 9620 high-performance automatic mercury porosimeter (Micromeritics, Norcross, GA, USA). The measurement conditions were as follows: 5 mL three-bulb penetrometer, mercury surface tension of 485 mN/m, advancing and receding contact angles both set at 130°, and a pressure range of 0.5–33,000 psia. The sample was subjected to intrusion-extrusion cycling in two stages: low pressure (0.5–30 psia) and high pressure (30–60,000 psia). The volume of mercury intruded at each equilibrium pressure was recorded. Based on the Washburn equation, the pressure data were converted into pore-size distributions, from which key structural parameters such as total pore volume, median pore diameter, porosity, and pore-size distribution curves were derived. The Washburn equation is given by (9):

(9) $$D=\frac{4\gamma cos\theta }{P}$$

D is the pore diameter (nm), γ is the surface tension of mercury (mN/m), θ is the contact angle (°), and P is the applied pressure (psia).

## 3. Results

### 3.1. Preparation Strategy and Physical Properties of MHCFP

**Figure 2:**
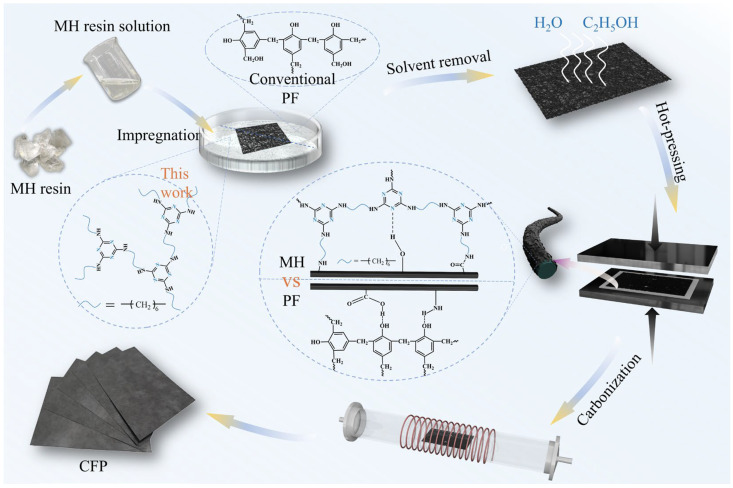
Flow chart of CFP fabrication.

MH resin is a thermosetting amino resin. To ensure effective chemical bonding between the resin and the carbon fibers, the carbon fiber preform was first impregnated and hot-pressed before carbonization. As shown in Figure 2, MH resin was dissolved in a 70% ethanol aqueous solution to prepare a low-viscosity impregnation solution, which facilitated thorough infiltration of the resin into the carbon fiber preform. The impregnated carbon fiber then underwent hot-pressing. During this process, a stepwise temperature and pressure increase strategy was employed to control the resin’s flowability in the early curing stage, thereby preventing premature resin extrusion that could lead to incomplete interfacial bonding or structural defects. Consequently, the resin is allowed to bond more firmly with the fiber surface during curing, forming a dense and uniform resin-fiber interface, which ensures the overall structural integrity and compactness of CFPs. The robust interfacial bonding established through this process effectively inhibited the propagation of microcracks induced by resin shrinkage during carbonization, enabling CFPs to maintain excellent mechanical strength and toughness.

**Figure 3:**
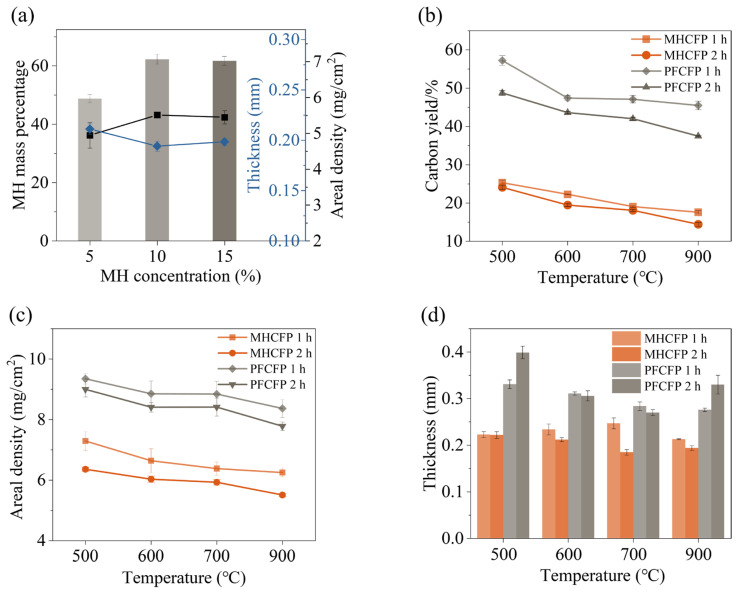
Comparison of the fundamental physical properties between MHCFP and PFCFP. (**a**) Effect of MH resin concentration on mass fraction of MH resin after hot-pressing, thickness, and areal density of carbon paper after carbonization. Variations of (**b**) char yield, (**c**) areal density, and (**d**) thickness of MHCFP and PFCFP with carbonization temperature and time.

In the fabrication of CFP, the binder concentration is a key parameter for tuning its microstructure and macroscopic properties [38]. This study systematically investigated the effect of different MH resin solution concentrations (5%, 10%, and 15%) at the same carbonization temperature and time (900 °C, 2 h) on the properties of CFPs (Figure 3a). Experimental results show that as the MH resin solution concentration increased from 5% to 10%, the mass fraction of MH resin in the hot-pressed carbon paper reached a maximum, accompanied by a significant increase in areal density and a decrease in thickness, leading to optimal overall performance. Further increasing the concentration to 15% resulted in a plateau in all performance parameters, with no significant changes observed. This can be attributed to the following: at a low concentration (5%), the resin was insufficient to fully infiltrate the carbon fibers and form an effective binding phase, resulting in a loose structure and inferior properties. At the optimal concentration (10%), the resin achieved complete infiltration and established a robust continuous phase between the fibers, thereby constructing a compact composite structure. When the concentration exceeded this threshold (>10%), the excess resin tended to be mechanically extruded under the high temperature and pressure of hot-pressing and could not be retained in the preform, thus failing to further enhance performance. The identification of this saturation threshold provides a crucial guideline for the fabrication of MH resin-based carbon paper.

Figure 3b–d presents the variations in thickness, areal density, and carbon yield of MHCFP and PFCFP with carbonization temperature and time. The areal density and char yield of both types of carbon paper decreased with increasing carbonization temperature and prolonging carbonization time, which is attributed to the continuous pyrolysis of the resin binder and the release of small molecules (e.g., H_2_O, NH_3_) at elevated temperatures. Notably, at the same carbonization temperature and time, the carbon yield of PF resin (37–57%) was significantly higher than that of MH resin (14–25%), resulting in a consistently higher areal density of PFCFP compared with MHCFP. In terms of thickness evolution, PFCFP showed a pronounced overall expansion, whereas MHCFP remained within a thin and stable range (0.19–0.25 mm). The expansion of PFCFP may stem from the debonding of its brittle resin-derived carbon from the fibers under thermal stress, leading to structural loosening. In contrast, the stable thickness of MHCFP suggests strong interfacial adhesion between its resin-derived carbon and the fibers, suppressing overall expansion during pyrolysis-induced shrinkage. The analysis of basic physical properties indicates that the MH resin system achieves structural stabilization under low carbon yield and low areal density, constructing a more robust composite skeleton with less solid residue.

**Figure 4:**
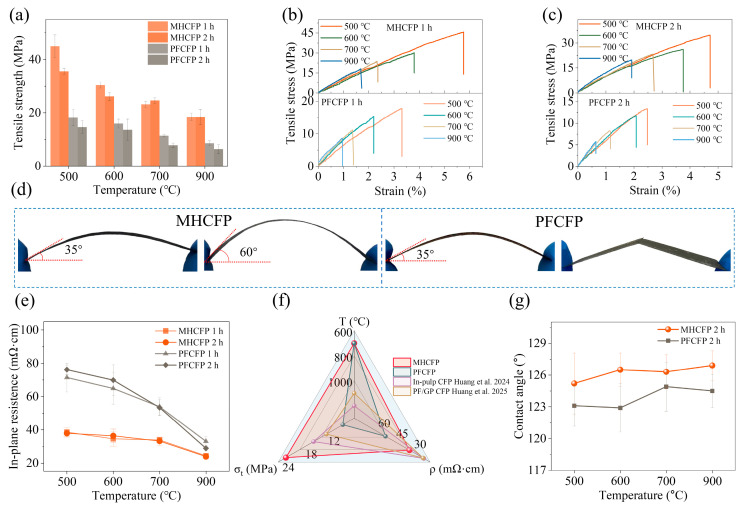
Comprehensive performance comparison of MHCFP and PFCFP under different carbonization temperatures and times. (**a**) Tensile strength of MHCFP and PFCFP under different carbonization temperatures and times; (**b**,**c**) tensile stress–strain curves of MHCFP and PFCFP at different carbonization temperatures with the same carbonization time (1 h, 2 h); (**d**) comparison of bending toughness between MHCFP-900 and PFCFP-900; (**e**) in-plane resistivity of MHCFP and PFCFP at different carbonization temperatures and time; (**f**) comparison of carbonization temperature (T), tensile strength (σₜ), and in-plane resistance (ρ) among MHCFP, PFCFP, and CFPs reported in the literature [38,39]; (**g**) water contact angles of MHCFP and PFCFP at different carbonization temperatures with the same carbonization time (2 h).

Figure 4 comprehensively compares the core properties of CFPs prepared using amino resin (MH) and traditional phenolic resin (PF) as binders. As illustrated, under identical preparation and carbonization conditions, MHCFP exhibits comprehensive advantages over PFCFP in mechanical, electrical, and surface properties, overcoming the heavy reliance of traditional carbon paper materials on high-temperature graphitization. Figure 4a demonstrates that the tensile strength of MHCFP across different carbonization temperatures (500–900 °C) is significantly higher than that of PFCFP, along with greater fracture strain (Figure 4b,c). A bending comparison test (Figure 4d) shows that PFCFP fractures when bent to 35 °, whereas MHCFP remains intact at 60 °, highlighting its combination of high strength and toughness. Figure 4e reveals that MHCFP achieves superior electrical conductivity even at relatively low carbonization temperatures, and its resistivity decreases gently with increasing temperature, demonstrating a low-temperature, high-efficiency characteristic. In contrast, the resistivity of PFCFP exhibits a marked reduction in resistivity only above 600 °C, underscoring its pronounced reliance on elevated carbonization temperatures. Figure 4f demonstrates that MHCFP can attain excellent mechanical and electrical performance at significantly lower temperatures, matching or surpassing conventional CFPs that typically require high-temperature carbonization [38,39]. Figure 4g shows that the water contact angle of MHCFP (125–127°) remains stably higher than that of PFCFP (123–125°), indicating an inherently stable hydrophobic nature. Such a pronounced performance difference must originate from fundamental distinctions in thermal behavior, microstructure, crystallographic characteristics, and surface chemical states between the two materials.

### 3.2. Characterizations of MHCFP

**Figure 5:**
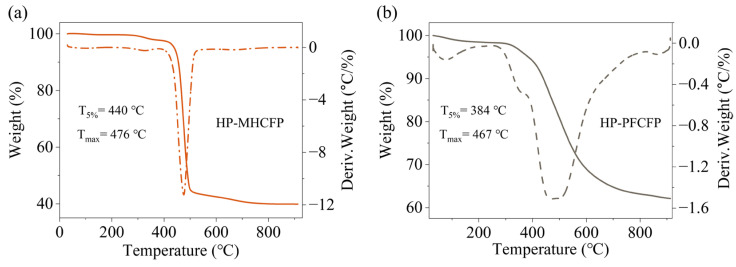
TG (solid line) and DTG (dotted line) curves of (**a**) HP-MHCFP and (**b**) HP-PFCFP. Abbreviations: T_5%_, the initial decomposition temperature; T_max_, the temperature of maximum decomposition rate.

Thermogravimetric analysis (Figure 5) demonstrates that the initial decomposition temperature (T_5%_ = 440 °C) of HP-MHCFP is approximately 56 °C higher than that of HP-PFCFP (vs. T_5%_ = 384 °C), and its temperature at the maximum weight loss rate (Tₘₐₓ = 476 °C) is likewise higher (Figure 5a,b). These results indicate that HP-MHCFP possesses significantly superior overall thermal stability relative to HP-PFCFP. As shown in Figure 5a, the sharper derivative thermogravimetry (DTG) peak of HP-MHCFP suggests that its major mass loss occurs over a narrower temperature range, reflecting a more homogeneous internal structure. This structural uniformity mitigates localized stress concentrations during the carbonization process, thereby promoting the formation of a continuous and well-integrated network architecture, which provides a favorable thermodynamic basis for its superior performance. Conversely, the broader and earlier decomposition peak exhibited by HP-PFCFP in Figure 5b points to greater structural heterogeneity and lower stability, which are prone to inducing stress concentration and microcrack formation, ultimately leading to performance deterioration.

**Figure 6:**
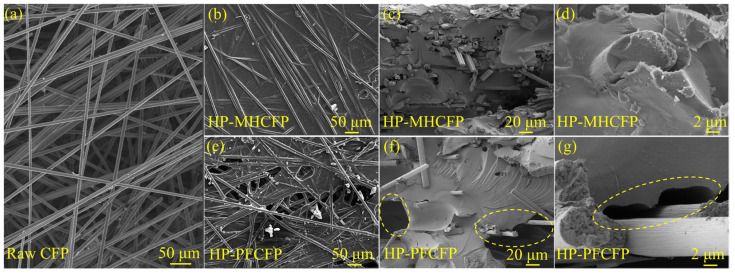
SEM images of (**a**) carbon fiber preform paper, (**b**,**e**) surface, and (**c**,**d**,**f**,**g**) cross-sectional views of HP-MHCFP and HP-PFCFP.

Figure 6a,b, reveals that, compared to the original carbon paper, MH resin forms a uniform and dense coating on the carbon fibers after hot-pressing, resulting in a composite structure free of interfacial gaps. In contrast, PF resin exhibits non-uniform and localized adhesion (Figure 6e). The cross-sectional views further illustrate the internal structural differences. For HP-MHCFP (Figure 6c,d), the cross-section shows that the carbon fibers are closely packed with a uniform distribution of the resin-derived phase, and there are no obvious large-scale interfacial gaps or delamination—consistent with the dense coating observed on the surface. For HP-PFCFP (Figure 6f,g), the cross-section reveals the presence of obvious discontinuities in resin distribution, along with noticeable interfacial gaps around the carbon fibers, reflecting discontinuous resin distribution and weak fiber–matrix bonding. The uniform and dense wrapping of carbon fibers by MH resin facilitates the formation of a continuous and integrated conductive carbon network during subsequent carbonization, enabling effective load transfer to the carbon fibers. This provides a structural foundation for the superior electrical conductivity and mechanical performance of MHCFP.

**Figure 7:**
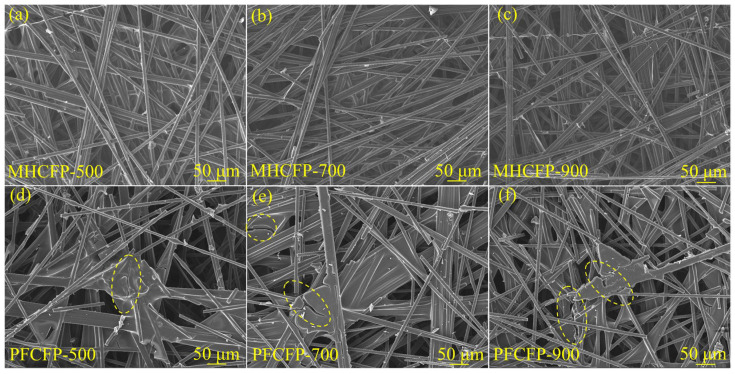
SEM images of (**a**–**c**) MHCFP and (**d**–**f**) PFCFP carbonized for the same duration (2 h) at different temperatures.

After carbonization, the structure of MHCFP evolves into a continuous three-dimensional network characterized by uniform shrinkage of the resin-derived carbon and tight bonding with the fibers, along with well-ordered pore distribution (Figure 7a–c). In comparison, Figure 7d–f demonstrates that although PFCFP retains a greater amount of resin-derived carbon, brittle shrinkage of the carbonized resin generates numerous cracks and causes interfacial debonding from the fibers, leading to a loose and defective two-phase separated structure. This fundamental difference in morphology—a dense, integrated structure versus a loose, fractured one—directly explains their divergent macroscopic properties: the intact interface in MHCFP ensures efficient load transfer and electron conduction, which is a prerequisite for the achievement of high strength, high toughness, and high conductivity at low carbonization temperatures; whereas the structural defects in PFCFP are primarily responsible for stress concentration and severely hindered charge transport, resulting in its inferior mechanical properties and difficulty in achieving high electrical conductivity.

**Figure 8:**
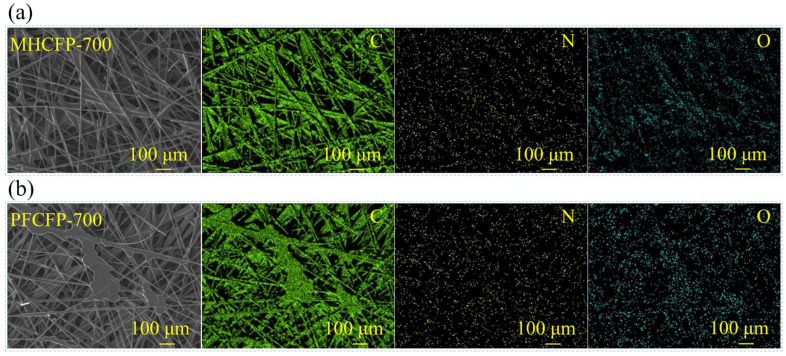
(**a**) SEM-EDS images of MHCFP, (**b**) SEM-EDS images of PFCFP.

SEM–EDS mapping further illustrates the differences in resin distribution between the two CFPs. In the case of MHCFP (Figure 8a), the C element signal clearly outlines a complete and continuous carbon fiber network, with a uniform intensity distribution along the direction of individual fibers. This indicates that the MH resin-derived carbon does not exist in the form of discrete agglomerates but rather is uniformly and densely coated on the surface of each carbon fiber, forming a continuous overlayer. This coating reinforces the fibers while largely preserving the open and interconnected porous framework of the original carbon fiber preform paper. In contrast, for PFCFP (Figure 8b), although the carbon fiber network remains discernible, the C element distribution shows greater heterogeneity: the contours of some fibers appear swollen due to accumulated resin-derived carbon, and distinct patchy C signals are present. This directly reveals that the phenolic resin-derived carbon fails to uniformly coat the fibers, instead partially filling the pores or accumulating on the fiber surfaces in the form of uneven blocks or clusters. Such non-uniform carbon distribution creates localized stress points within the structure, which is the direct microstructural origin of the deteriorated pore structure and reduced mechanical properties.

**Figure 9:**
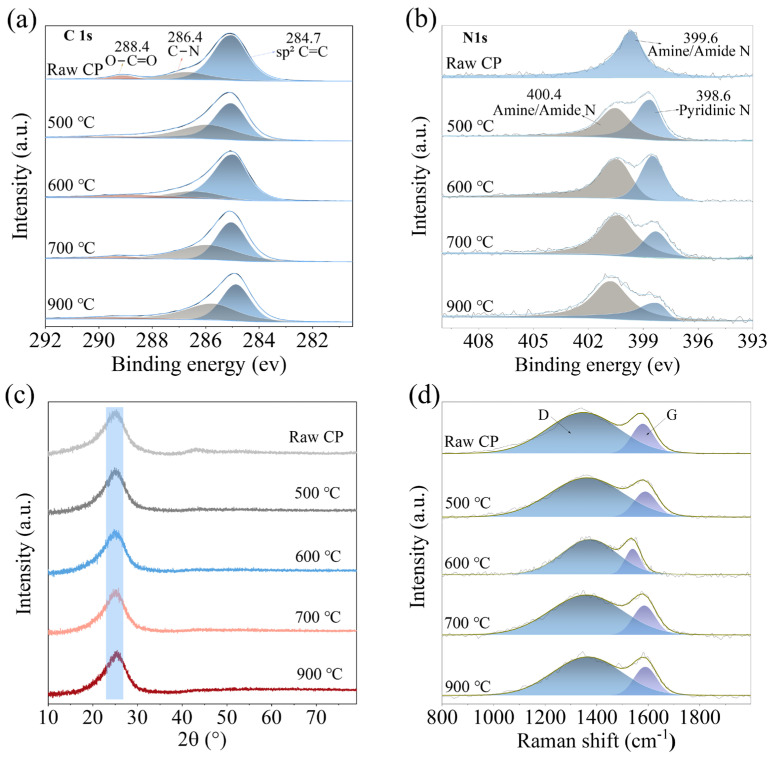
XPS spectra of (**a**) C 1s and (**b**) N 1s regions obtained for Raw CFP and MHCFP at different carbonization temperatures. (**c**) XRD and (**d**) Raman patterns of Raw CFP and MHCFP at different carbonization temperatures. (G peak, Represents the degree of order and structural integrity of carbon materials. D peak: Represents defects and disordered structures within carbon materials.)

To further elucidate the physicochemical origins of the performance differences between MHCFP and PFCFP, surface elemental and functional group analyses were conducted on Raw CFP and MHCFP. The elemental composition data in Table 1 show that the surface of Raw CFP contains 11.3 at% oxygen and 3.7 at% nitrogen. Combined with the presence of –O–C=O and –NH_2_ signals in the C 1s and N 1s spectra (Figure 9a,b), these results indicate the existence of carboxyl and amino functional groups on the fiber surface, which provide reactive sites for chemical bonding with the resin. When PF resin serves as the binder, its hydroxymethyl functional groups rapidly undergo bulk self-condensation during the curing process. This reaction overwhelmingly dominates and suppresses potential chemical interactions (e.g., esterification or aminomethylation) with the fiber surface. As a result, adhesion between the resin and fibers relies primarily on hydrogen bonding. During pyrolysis, mismatched thermal expansion coefficients and volume shrinkage between the two phases induce stress concentration at the interface, leading to microcrack formation (Figure 7d–f), ultimately resulting in a loose structure and poor mechanical properties of the carbon paper. In contrast, the MH resin is rich in highly reactive amino groups. During the hot-pressing curing process, these amino groups undergo self-condensation while efficiently reacting with the functional groups on the carbon fiber surface, thereby forming a robust interfacial bond. Such robust interfacial bonding effectively suppresses interfacial debonding and, consequently, maintains interfacial integrity even during carbonization, when resin shrinkage occurs. The intact interface allows the resin-derived carbon to uniformly and densely coat the fiber surface. (Figure 6b and Figure 7a–c). Such covalent-bond-based interfacial integration is the origin of the exceptional tensile strength, flexibility, and integrity of the conductive network in MHCFP.

The resistivity evolution shown in Figure 4e aligns with the structural ordering process revealed by XPS, XRD, and Raman spectroscopy in Figure 9, collectively elucidating the fundamental differences in conduction mechanisms between MHCFP and PFCFP. Although increasing the carbonization temperature reduces the carbon yield and decreases the density of conductive pathways due to resin pyrolysis, the overall resistivity of both materials continues to decline. This indicates that the enhancement in the intrinsic conductivity of the carbon skeleton plays a dominant role.

For MHCFP, as the carbonization temperature increased from 500 °C to 900 °C, its resistivity showed a gradual decreasing trend. High-resolution XPS in Figure 9a,b analysis revealed the structural evolution mechanism at the atomic chemical-state level. The C 1s spectra of all samples (Figure 9a) exhibited a main peak near 284.6 eV, along with the asymmetric peak shape characteristic of an sp^2^-hybridized carbon network, confirming that an sp^2^ carbon framework with a delocalized π-electron structure had already formed during the low-temperature carbonization process [40]. However, no prominent π–π* shake-up satellite peak was observed around 290.5 eV, indicating that the conjugation scale and in-plane ordering of the formed sp^2^ carbon network had not reached a highly crystalline graphite state. The XPS spectrum of MHCFP (Figure 9b) exhibits characteristic peaks corresponding to pyridinic nitrogen (N) at 398.6 eV and amine/amide nitrogen at 400.4 eV. The signal at 398.6 eV confirms the partial retention of the triazine-ring aromatic framework derived from the MH resin during pyrolysis. In the initial stage of pyrolysis, this rigid structural unit acts as a locally ordered template for carbon atom rearrangement, effectively guiding the low-temperature formation of sp^2^ carbon clusters, which is crucial for the low-temperature ordering achieved in MHCFP. Meanwhile, the amine/amide nitrogen at 400.4 eV originates from residual aliphatic amine structures derived from hexamethylenediamine. This type of nitrogen enhances the local electron density of the carbon network through an electron-donating effect, thereby optimizing charge carrier transport pathways. Additionally, nitrogen doping introduces additional charge carriers [41]. These effects synergistically optimized the carrier transport pathways and further enhanced the low-temperature conductivity of MHCFP.

This short-range ordered carbon structure is corroborated by both XRD and Raman spectroscopic results. The XRD patterns in Figure 9c show that all samples exhibit a broadened (002) diffraction peak around 2θ ≈ 26 °, indicating that MHCFP subjected to low-temperature carbonization already exists in a structural state characterized by the coexistence of short-range ordered sp^2^ carbon clusters and amorphous carbon [42,43]. Table 2 shows that as the carbonization temperature increases from 500 °C to 900 °C, the diffraction peak gradually sharpens and shifts to higher angles slightly. The interlayer spacing (d_002_) of sp^2^ carbon clusters decreases overall, and the stacking thickness (L_c_) increases from 1.30 nm to 1.46 nm. As shown in Figure 9d and Table 3, the proportion of the G peak in the Raman spectra gradually increases with rising carbonization temperature, corresponding to a significant increase in the in-plane crystallite size (L_a_) of sp^2^ carbon clusters from 2.83 nm to 3.23 nm, as calculated from the I_D_/I_G_ ratio. This trend is consistent with the increase in stacking thickness (L_c_) observed in XRD, jointly confirming the synergistic ordering of the sp^2^ carbon network in both in-plane and out-of-plane directions. These results clearly demonstrate that the MH resin system can drive significant structural ordering of the resin-derived carbon at moderate temperatures of 500–900 °C, guided by its intrinsic triazine ring architecture. It is precisely this rapidly formed and continuously optimized conductive network of sp^2^ carbon clusters at low temperatures that enables MHCFP to achieve exceptional electrical conductivity without undergoing conventional high-temperature graphitization.

The conductive pathways, synergistically constructed by short-range ordered sp^2^-carbon networks and nitrogen doping, enable MH-derived carbon to establish an excellent conductive foundation without undergoing extreme thermal treatment. In contrast, the resistivity of PFCFP shows a monotonic and significant decrease as the carbonization temperature rises from 500 °C to 900 °C. The improvement in its conductivity relies entirely on high-temperature-driven ordering of the carbon microcrystals, reflecting the lack of low-temperature structural-directing capability in the PF resin.

The ordered sp^2^-hybridized carbon framework in MHCFP not only enhances electrical conductivity but also significantly improves surface wetting behavior, owing to its inherent high chemical inertness and low polarity. According to surface energy theory, such a surface exhibits notably reduced surface energy and a weakened polar component (γ^P^) [44]. The Young’s equation indicates that the wetting behavior of a solid surface is directly governed by its surface energy and polar component. Therefore, a combination of low surface energy and a low polar component constitutes the key physicochemical basis for achieving a high-water contact angle [45]. Consequently, the ordered sp^2^-carbon framework in MHCFP not only constructs an efficient conductive network but also imparts stable and excellent hydrophobic properties (water contact angle > 125°), owing to its low surface energy and polarity.

### 3.3. Characterization of Pore Structure and Analysis of Gas Transport Performance in MHCFP

**Figure 10:**
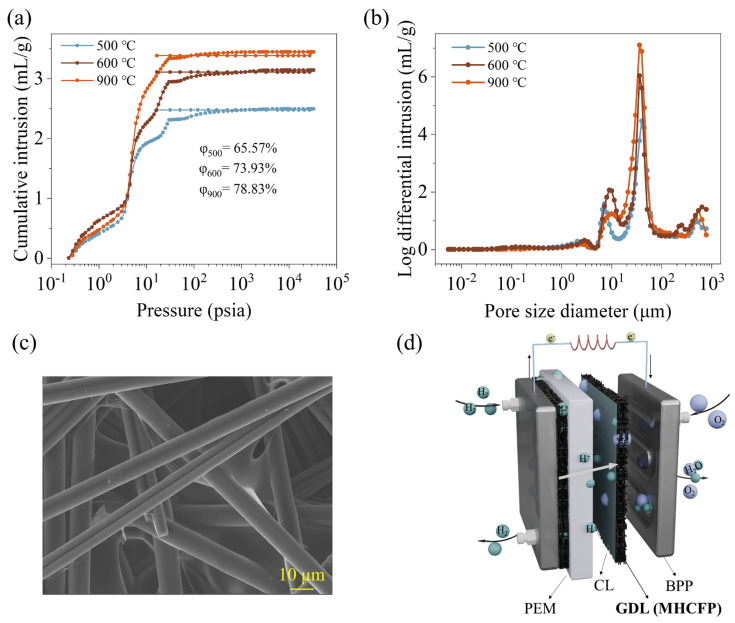
(**a**) Mercury intrusion curves and (**b**) pore-size distribution curves of MHCFP carbonized for the same duration (2 h) at different temperatures; (**c**) SEM images of MHCFP-900. (**d**) Schematic illustration of the functional roles of MHCFP as a gas diffusion layer in a proton exchange membrane fuel cell (PEMFC). Abbreviations: φ, porosity; PEM, proton exchange membrane; CL, catalyst layer; GDL, gas diffusion layer; BPP, bipolar plate.

Figure 10a shows that the mercury intrusion volume of MHCFP levels off rapidly at relatively low pressure (<100 psia), indicating a well-interconnected, three-dimensional open-pore network. As the carbonization temperature increases from 500 °C to 900 °C, the total intruded mercury volume (i.e., total pore volume) increases significantly, corresponding to a rise in porosity from 65.57% to 78.83%. This increase is attributed to the shrinkage of the resin-derived carbon during pyrolysis, which generates additional secondary pores. The pore-size distribution curve (Figure 10b) reveals that the pores in MHCFP are mainly concentrated in the macro-pore range of 10–100 μm, indicating that the material is dominated by large-diameter pore structures. According to porous-media transport theory [46], pores within this size range facilitate gas transport in the viscous-flow regime with low flow resistance, thus enabling efficient gas transport. Combined with SEM observations (Figure 10c), the carbon fibers are uniformly and densely coated by the carbon derived from MH-resin pyrolysis, which ensures smooth pore-channel walls that are beneficial for gas-phase transport and inhibit the formation of liquid-water films.

The three-dimensional network constructed by MHCFP, featuring high porosity, predominantly large pore size, good interconnectivity, and smooth pore walls, highly matches the core requirements of gas diffusion layers (GDLs) for proton exchange membrane fuel cells (PEMFCs) in terms of mass transport. The continuous large-pore channels provide efficient pathways for reactant gases (H_2_/O_2_) to permeate to the catalyst layer while also facilitating the removal of generated water vapor and liquid water to the flow field. Its hydrophobic surface (contact angle > 125°) and smooth pore walls act synergistically to effectively reduce the risk of flooding. As shown in the schematic diagram in Figure 10d, the structural characteristics of MHCFP fully meet the requirements for balanced transport of gas, electrons, and water within PEMFCs, thereby ensuring its high-efficiency and stable operation. In future research, we will attempt to process the MHCFP into a complete gas diffusion layer material and test its application potential in real fuel cell scenarios.

## 4. Conclusions

This study systematically elucidates the multiscale synergistic mechanism responsible for the comprehensive performance breakthrough of MHCFP based on a novel amino resin (MH) binder under moderate low-temperature carbonization conditions. The amino functional groups in the MH resin react with reactive groups on the carbon fiber surface, establishing a robust interfacial structure that imparts MHCFP with high tensile strength (18–45 MPa), significantly higher than that of PFCFP (6–18 MPa). The triazine ring structures in the MH resin guide the formation of sp^2^ carbon clusters at relatively low carbonization temperatures, while the doping of amine/amide nitrogen introduces additional charge carriers, enabling MHCFP to achieve much lower in-plane electrical resistivity (24–39 mΩ·cm) than PFCFP (29–83 mΩ·cm) even under low carbonization temperatures (500–900 °C). The low surface energy and low polarity components of the sp^2^-hybridized carbon framework confer upon MHCFP superior hydrophobicity (125–127°) compared to PFCFP (123–125°). More importantly, the system simultaneously constructs a highly interconnected three-dimensional network dominated by macropores (10–100 μm) and possessing a porosity exceeding 78%, providing a solid structural foundation for efficient water–gas management. This multiscale design strategy, integrating strong interfacial bonding, low-temperature-induced formation of sp^2^ carbon clusters, and high-porosity network construction, successfully resolves the core trade-off among mechanical properties, electrical conductivity, and mass transport capability inherent in traditional phenolic resin-based CFP. MHCFP demonstrates great potential to replace conventional phenolic resin-based CFP, offering a highly promising material solution for the development of next-generation fuel cell gas diffusion layers with both high performance and low energy consumption.

## Figures and Tables

**Table 1:** Surface compositions of different samples determined by XPS.

Sample	Element Content (wt%)			N/C
	C	N	O	
Raw CFP	84.96	3.75	11.29	0.044
MHCFP-500	81.34	11.09	7.57	0.14
MHCFP-600	82.14	8.76	8.01	0.11
MHCFP-700	85.97	5.57	8.29	0.065
MHCFP-900	87.66	4.43	7.91	0.051

**Table 2:** Structural parameters of different samples derived from XRD spectra.

Sample	2θ/°	FWHM/°	d_002_/nm	L_c_/nm
Raw CFP	24.78	6.89	0.3595	1.17
MHCFP-500	25.01	6.23	0.3561	1.30
MHCFP-600	24.94	6.21	0.3571	1.30
MHCFP-700	25.00	6.18	0.3563	1.31
MHCFP-900	25.01	5.57	0.3561	1.46

**Table 3:** Structural parameters of different samples derived from Raman spectra.

Sample	I_D_/a.u.	I_G_/a.u.	I_D_/I_G_	L_a_/nm	X_G_/%
Raw CFP	143.05	103.71	1.379	3.15	42.03
MHCFP-500	35.81	23.30	1.537	2.83	39.42
MHCFP-600	20.40	14.98	1.362	3.19	42.34
MHCFP-700	23.22	17.06	1.361	3.20	42.35
MHCFP-900	22.23	16.52	1.345	3.23	42.36

## Data Availability

The original contributions presented in this study are included in the article. Further inquiries can be directed to the corresponding authors.
